# Hepatic Glucose Output Inhibition by Mexican Plants Used in the Treatment of Type 2 Diabetes

**DOI:** 10.3389/fphar.2020.00215

**Published:** 2020-03-03

**Authors:** Gerardo Mata-Torres, Adolfo Andrade-Cetto, Fernanda Artemisa Espinoza-Hernández, René Cárdenas-Vázquez

**Affiliations:** ^1^Laboratorio de Etnofarmacología, Facultad de Ciencias, Universidad Nacional Autónoma de México, Mexico City, Mexico; ^2^Laboratorio de Biología Animal Experimental, Facultad de Ciencias, Universidad Nacional Autónoma de México, Mexico City, Mexico

**Keywords:** medicinal plant, hepatic glucose output, type 2 diabetes, glucose 6 phosphatase, traditional medicine

## Abstract

*De novo* hepatic glucose production or hepatic gluconeogenesis is the main contributor to hyperglycemia in the fasting state in patients with type 2 diabetes (T2D) owing to insulin resistance, which leads to at least twice as much glucose synthesis compared to healthy subjects. Therefore, control of this pathway is a promising target to avoid the chronic complications associated with elevated glucose levels. Patients with T2D in the rural communities of Mexico use medicinal plants prepared as infusions that are consumed over the day between meals, thus following this rationale (consumption of the infusions in the fasting state), one approach to understanding the possible mechanism of action of medicinal plants is to assess their capacity to inhibit hepatic glucose production. Furthermore, in several of these plants, the presence of phenolic acids able to block the enzyme glucose-6-phosphatase (G6Pase) is reported. In the present work, extracts of *Ageratina petiolaris*, *Bromelia karatas*, *Equisetum myriochaetum*, *Rhizophora mangle*, and *Smilax moranensis*, which are Mexican plants that have been traditionally used to treat T2D, were assayed to evaluate their possible hepatic glucose output (HGO) inhibitory activity with a pyruvate tolerance test in 18-h fasted STZ-NA Wistar rats after oral administration of the extracts. In addition, the *in vitro* effects of the extracts on the last HGO rate-limiting enzyme G6Pase was analyzed. Our results showed that four of these plants had an effect on hepatic glucose production in the *in vivo* or *in vitro* assays. *A. petiolaris* and *R. mangle* extracts decreased glucose output, preventing an increase in the blood glucose levels and sustaining this prevented increase after pyruvate administration. Moreover, both extracts inhibited the catalytic activity of the G6Pase complex. On the other hand, even though *S. moranensis* and *B. karatas* did not exhibit a significant *in vivo* effect, *S. moranensis* had the most potent inhibitory effect on this enzymatic system, while the *E. myriochaetum* extract only inhibited hepatic glucose production in the pyruvate tolerance test. Because of the traditional method in which diabetic patients use plants, hepatic glucose production inhibition seems to be a mechanism that partially explains the common hypoglycemic effect. However, further studies must be carried out to characterize other mechanisms whereby these plants can decrease HGO.

## Introduction

Diabetes is a chronic condition that occurs when the body cannot produce enough insulin or respond properly to the insulin it does produce. In type 2 diabetes (T2D), the inability of the cells to respond to low levels of insulin, defined as insulin resistance, leads to elevated glucose levels in the bloodstream, or hyperglycemia (International Diabetes Federation, 2017). In the long term, uncontrolled hyperglycemia induces the development of complications (neuropathy, nephropathy, and retinopathy, among others) that impair the quality of life of individuals with T2D.

Hyperglycemia in T2D, among other factors, is caused by an increased hepatic glucose output (HGO), which is triggered by insulin resistance in the fasting state. Poor insulin signaling in the liver cannot efficiently suppress HGO. Overall, hyperglycemia is the sum of two glucose inputs, one from the gastrointestinal tract at the postprandial stage, and another from endogenous production. The liver produces approximately 85% of endogenous glucose, and half of this comes from gluconeogenesis. In a healthy subject, the production rate is approximately 1.8–2.0 mg/kg/min after overnight fasting. On the other hand, this rate increases at least two-fold in patients with T2D due to impaired gluconeogenesis (Cersosimo et al., 2018). Furthermore, when the balance between glucose production and glucose storage is disrupted, as observed in patients with T2D, glucose homeostasis is altered and significantly contributes to hyperglycemia (Sharabi et al., 2015). Some of the identified mechanisms responsible for the increased rate of gluconeogenesis include high circulating levels of gluconeogenic precursors such as lactate, pyruvate, alanine, and glycerol; increased free fatty acid oxidation; enhanced sensitivity to glucagon; and decreased sensitivity to insulin (Cersosimo et al., 2018).

Gluconeogenesis is an anabolic pathway that plays a major role in glucose metabolism by maintaining the glucose demand of the organs during starvation and after a meal high in fat and protein without carbohydrates. Gluconeogenesis can be regulated depending on the energy demand of the organism by activation or inhibition of the rate-limiting enzymes at different levels, such as substrate delivery, mass action regulation, allosteric activation, covalent modification, and alteration of gene expression. Among these enzymes, glucose-6-phosphatase (G6Pase) can control hyperglycemia because it determines the production of glucose released from gluconeogenesis and glycogenolysis (HGO) (Jawad et al., 2016). This enzyme is a multifunctional system attached to the membrane of the endoplasmic reticulum (ER) formed by three specific translocases (T1, T2, and T3) whose functions are: T1, glucose-6-phosphate (G6P) influx transport into the ER; T2, phosphate efflux transport; and T3, free glucose efflux transport; and a phosphatase subunit, in accordance with the widely accepted “substrate-transport” model (van Schaftingen and Gerin, 2002).

Inhibition of the rate-limiting enzymes in gluconeogenesis by phytochemicals as a target to treat T2D has attracted attention in recent years. Medicinal plants could provide new therapeutic compounds that allow regulation of gluconeogenic enzymes at different levels (Andrade-Cetto, 2012). For example, metformin, a first-line drug prescribed for T2D management derived from the medicinal plant *Galega officinalis* L. (Fabaceae) (French lilac or Goat’s rue) (Bailey, 2017), can decrease hepatic gluconeogenesis flux by reducing the gene expression of G6Pase and phosphoenolpyruvate carboxykinase (PEPCK), and promoting allosteric inhibition of fructose-1,6-bisphosphatase (F-1,6-Pase) (Hardie, 2013; Viollet and Foretz, 2013; Foretz et al., 2014; Tan et al., 2016; Hunter et al., 2018). Moreover, chlorogenic acid (CA), which is the most abundant isomer of caffeoylquinic acid present in foods, such as coffee and green tea, has been identified as a reversible competitive inhibitor of G6Pase T1 translocase (Hemmerle et al., 1997; Charkoudian et al., 2012; Naveed et al., 2018). This phenolic acid is the most studied natural compound found in a wide variety of plant species that has been associated with improved both glucose tolerance and insulin resistance in animal models (Meng et al., 2013).

To assess the potential inhibitory effects of phytochemicals on HGO, fasting hyperglycemic animal models are needed. The STZ-NA hyperglycemic model developed by Masiello et al. (1998), which consists of generating a “type-2-diabetogenic” syndrome with the ability to respond to glucose-stimulated insulin secretion, is characterized by stable hyperglycemia due to the partial protection of nicotinamide (NA) against the specific β-cytotoxic effect of streptozotocin (STZ). Although this model lacks insulin resistance (Szkudelski, 2012), the decreased insulin secretion due to the residual β-cells is responsible for the reduced glucose tolerance in these induced organisms (Szkudelski et al., 2013). Moreover, since β-cells are able to respond to drugs, this model has been used to assess the potential glucose-lowering effects of natural products, which are always evaluated after a fasting period (Eddouks et al., 2012).

In Mexico, type 2 diabetic patients use medicinal plants together with the prescribed medication to control glucose levels (Andrade-Cetto and Heinrich, 2005). As a result of our fieldwork performed in some regions of Mexico, we select five relevant medicinal plants traditionally used for the treatment of the illness. In brief, in the town of “Nopala” in Oaxaca state, diabetic patients use an infusion of the roots from *Smilax moranensis* M. Martens & Galeotti (Smilacaceae, known as “Cocolmecatl”) as well as the infusion of the bark from *Rhizophora mangle* L. (Rhizophoraceae, known as “Mangle Rojo”) to control the disease. In the state of Hidalgo, in the towns of “Tlanchinol” and “Tamala,” the patients use both the infusions of the aerial parts of *Bromelia karatas* L. (Bromeliaceae, known as “piñuela”) and *Equisetum myriochaetum* Schltdl. & Cham. (Equisetaceae, known as “cola de caballo”) which were highly recommended by the traditional healer “Isabel Escalante, RIP” to treat T2D. In Mexico City, in the “Sonora” market of medicinal plants, the sellers suggest the disease control by using an infusion of the aerial parts of *Ageratina petiolaris* (Moc. ex DC.) R.M. King & H. Rob. (Asteraceae, traditionally known by its Spanish name “hierba del ángel,” or its Nahuatl name “Yolochichotl”) which is in part brought to market from “Tenancingo,” Mexico State.

In previous works, we analyzed some aspects of their hypoglycemic effect as well as the phytochemical composition (Andrade-Cetto et al., 2000; Wiedenfeld et al., 2000; Andrade-Cetto, 2011a; Andrade-Cetto and Rubalcaba-Mares, 2012; Andrade-Cetto and Medina-Hernández, 2013; Bustos-Brito et al., 2016) for some of these plants. We also assessed the chronic hypoglycemic effect, the α-glucosidase inhibition or the insulin secretory effect (Revilla et al., 2002; Andrade-Cetto et al., 2008, 2015, 2017; Escandón-Rivera et al., 2019; Romo-Pérez et al., 2019). In these studies, we noticed that the hypoglycemic effect of a plant cannot be attributed to a single factor but to the combination of different mechanisms.

In the field, we documented that these plants are used as an infusion that diabetic patients usually drink over the course of a day in the so-called preparation “agua de uso.” Since the infusion is consumed during the fasting state, it is a rational that a possible mechanism contributing to the hypoglycemic effect of the plants can be related to HGO inhibition. To test this hypothesis, we performed pyruvate tolerance tests in fasting STZ-NA hyperglycemic rats *in vivo* and evaluated the ability of these plants to inhibit G6Pase *in vitro.*

## Materials and Methods

### Plant Extracts

In previous works, two kinds of plant extracts, aqueous (like the traditional infusion) and ethanol-water, were tested for their hypoglycemic effects. In the present study, we selected the extracts and doses that previously presented better biological activity (phytochemical profiles and voucher numbers are provided as Supplementary Material): ethanol-water extracts from *R. mangle* (bark, collected in “Manialtepec, Oaxaca, Mexico”) and *S. moranensis* (roots, collected in “Nopala, Oaxaca, Mexico”), aqueous extracts from *A. petiolaris* (aerial parts, collected in “Tenancingo, Estado de Mexico, Mexico”), *B. karatas*, and *E. myriochaetum* (both aerial parts, collected in “Tamala, Hidalgo, Mexico”). The ethanol-water extracts were prepared with 20 g of the plant in 500 ml of a mixture of ethanol:water (1:1) at 40°C for 4 h with agitation. The ethanol was removed on a Büchi rotatory evaporator, and the aqueous portion was frozen at −40°C. The water was eliminated by sublimation with a Labconco freeze dryer at reduced pressure. Aqueous extracts were made by boiling 20 g of the plant in 500 ml of water for 15 min with agitation. Afterward, the extracts were frozen at −40°C, and the water was finally removed by sublimation.

### Hyperglycemic Animals

Eight-week-old Wistar rats were obtained from the bioterium of the School of Sciences, National Autonomous University of Mexico (UNAM). Animals were maintained with free access to food and water in a room at 25°C and 55% humidity under 12:12 h light:dark periods.

Hyperglycemia was induced as described previously (Masiello et al., 1998). Briefly, overnight fasting rats were administered 65 mg/kg i.v. injection of STZ (Sigma-Aldrich S0130) in 0.1 M acetate buffer, pH 4.5, and 15 min later they received a 150 mg/kg i.p. injection of NA (Sigma-Aldrich). One week later, the animals with fasting glucose values over 180 mg/dl were selected to perform pyruvate tolerance tests.

This study was carried out in accordance with the principles of the Basel Declaration and recommendations of the Committee for the Update of the Guide for the Care and Use of Laboratory Animals (National Research Council, 2011). The protocol was approved by the Committee of Academic Ethics and Scientific Responsibility (CEARC) of the Faculty of Science, UNAM (PI_2020_01_001).

### Pyruvate Tolerance Test

Wistar rats fasted for 18 h were administered with vehicle (saline), metformin (Roche^®^) or extracts by gavage. Fifteen minutes later, vehicle or sodium pyruvate (2 g/kg bw, Sigma-Aldrich) were injected intraperitoneally. Blood samples were taken from the tail vein, and glucose levels were measured just before pyruvate administration (time 0) and 30, 60, 90, and 120 min later by using an Accutrend Plus (Roche^®^) glucometer. The animals were assigned into ten experimental groups (*n* = 6 per group): normoglycemic (N); normoglycemic + pyruvate (NP); hyperglycemic (H); hyperglycemic + pyruvate (HP); hyperglycemic + pyruvate + metformin 500 mg/kg (HPM); hyperglycemic + pyruvate + *A. petiolaris* 160 mg/kg (HPAp); hyperglycemic + pyruvate + *B. karatas* 218 mg/kg (HPBk); hyperglycemic + pyruvate + *E. myriochaetum* 330 mg/kg (HPEm); hyperglycemic + pyruvate + *R. mangle* 90 mg/kg (HPRm) and hyperglycemic + pyruvate + *S. moranensis* 80 mg/kg (HPSm).

### Liver Microsome Isolation

Four overnight-fasted Wistar rats were anesthetized with pentobarbital (6 mg/100 g b.w., i.p.). Livers were dissected and homogenized in a 7 ml Dounce tissue grinder to obtain a 20% homogenate in buffer (0.25 M sucrose, 1 mM EDTA, 5 mM HEPES, pH 7.4). The homogenate was filtered through a nylon mesh and submitted to differential centrifugation as described previously (Andrade-Cetto and Cárdenas-Vázquez, 2010). The 100,000 *g* × 1 h pellets were stored at −40°C until use.

### Glucose-6-Phosphatase Assay

A colorimetric assay was performed to assess microsomal G6Pase inhibition by the extracts as described previously (Andrade-Cetto and Cárdenas-Vázquez, 2010; Andrade-Cetto, 2011b). The test consists in the addition, from least to greatest, several concentrations of the potential inhibitor in the assayed medium which contains intact rat hepatic microsomes. The reaction starts by supplementing the substrate, while the stop solution, which incorporates sodium molybdate and ascorbic acid, is added at the end. Therefore, a reduced blue phosphomolybdate complex is formed due to the presence of the released inorganic phosphate which is proportional to the enzymatic activity. In brief, in 100 μl of total assay volume, buffer (40 mM imidazole, 0.25 M sucrose, pH 7), 20 mM G6P, microsomes and CA or plant extracts at different concentrations were added. The reaction was started by the addition of G6P, incubated at 22°C for 20 min and stopped with the addition of 900 μl of a solution containing 0.42% ammonium molybdate in 1 N H_2_SO_4_, 10% SDS and 10% ascorbic acid. After incubation of the media at 45°C for 20 min, the inorganic phosphate was quantified colorimetrically at 830 nm (Arion, 1989). Assays were performed by triplicate.

### Statistical Analysis

Pyruvate tolerance test data were expressed as the mean glucose level ± standard error (SEM) at each time point on the curve. Area under curve (AUC) data were expressed as (mg/dl) × min ± SEM. One-way ANOVA with Tukey’s *post hoc* tests were performed to compare glucose means among all the groups at each time point, the glucose means from each group versus the corresponding basal value, and the AUC values among the groups. *P*-values less than 0.05 were considered statistically significant. IC_50_ values were calculated by plotting concentration-response curves to find the best fitting regression model (linear or non-linear). Analysis was carried out in GraphPad Prism version 7.00 (GraphPad Software, La Jolla, CA, United States).

## Results

Phytochemical composition of the tested plants was previously reported by our group (see references above). For reference purposes, we have included the HPLC-DAD profiles of the tested extracts that can be consulted in Supplementary Material.

### *In vivo* Pyruvate Tolerance Tests

**FIGURE 1:**
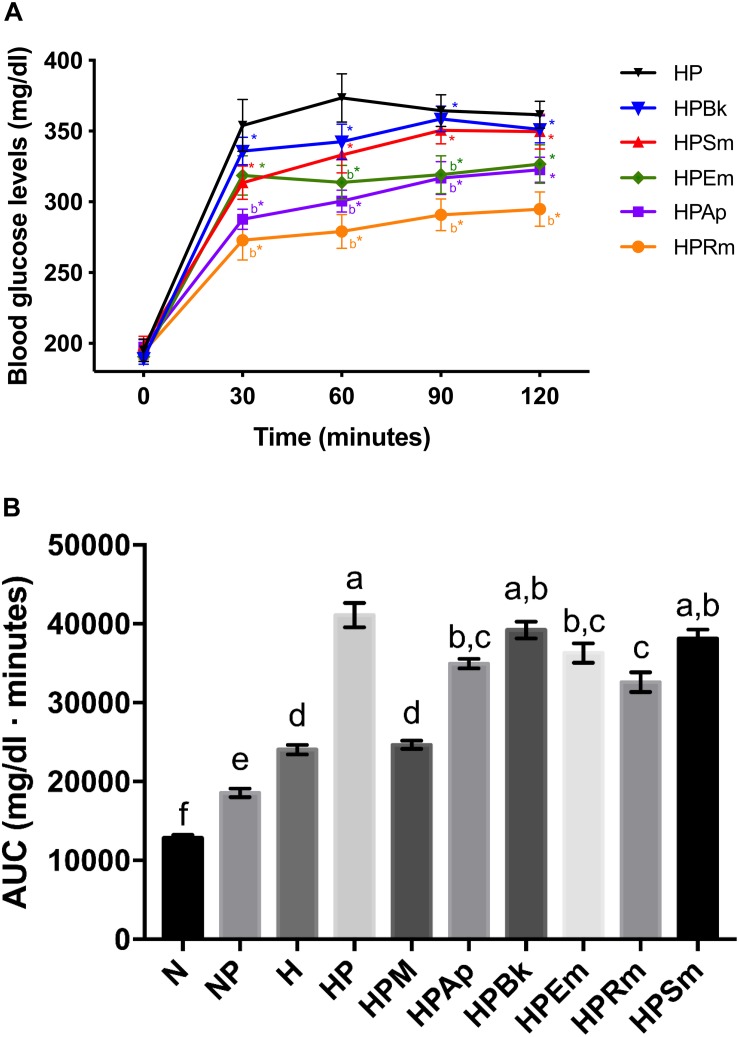
Effect of plant extracts on pyruvate tolerance tests on STZ-NA induced hyperglycemic rats. **(A)** Comparison of plant extract curves. Letter “b” indicates significant difference versus hyperglycemic + pyruvate group (HP) at *p* < 0.05; *indicates significant difference with time 0 of the same group at *p* < 0.05. **(B)** AUC values. The bars represent the mean ± SEM. Different letters over bars indicate statistically significant differences at *p* < 0.05 (a > b > c > d > e > f). N, normoglycemic group; NP, normoglycemic + pyruvate group; H, hyperglycemic; HP, hyperglycemic + pyruvate group; HPM, hyperglycemic + pyruvate + metformin group; HPAp, hyperglycemic + pyruvate + *Ageratina petiolaris* group; HPBk, hyperglycemic + pyruvate + *Bromelia karatas* group; HPEm, hyperglycemic + pyruvate + *Equisetum myriochaetum* group; HPRm, hyperglycemic + pyruvate + *Rhizophora mangle* group; HPSm, hyperglycemic + pyruvate + *Smilax moranensis* group.

To evaluate gluconeogenesis as the major HGO source, rats were deprived of food for 18 h to deplete hepatic glycogen storage. Next, pyruvate was administered as the substrate for the inhibition assessment. As shown in Table 1, both intraperitoneal injection and oral administration of vehicle to normoglycemic (N) and hyperglycemic (H) control rats did not affect the blood glucose levels over the 2-h period of analysis (N and H groups), while pyruvate injection significantly increased these levels, as observed in the NP and HP groups. However, the effect was different between these two groups, since in the NP group, blood glucose levels significantly increased by approximately 60 mg/dl (57%) between 30 and 60 min after pyruvate administration, and almost returned to basal values after 2 h; in the HP group, pyruvate injection raised glucose levels by approximately 160 mg/dl (81%), which remained higher compared to the control group (H) and did not return to the initial value. In other words, even though pyruvate administration significantly increased the overall glucose in normo- and hyperglycemic rats compared to their controls (Figure 1A), a differential effect over time was observed due to the physiological state of each group.

**TABLE 1:** Plasma glucose values measured at the pyruvate tolerance test on STZ-NA induced hyperglycemic rats.

**Glucose**	**T0**	**T30**	**T60**	**T90**	**T120**
**groups**	**(mg/dl)**	**(mg/dl)**	**(mg/dl)**	**(mg/dl)**	**(mg/dl)**
1. N	109 ± 4	111 ± 4	107 ± 3	104 ± 4	102 ± 3
	100%	102%	98%	96%	94%
2. NP	111 ± 3	173 ± 4^a^*	176 ± 7^a^*	149 ± 6*	132 ± 6
	100%	157%	158%	134%	119%
3. H	193 ± 4^a^	213 ± 7^ab^	200 ± 5^ab^	198 ± 7^ab^	189 ± 4^ab^
	100%	110%	104%	103%	98%
4. HP	195 ± 8	354 ± 18*	373 ± 17*	365 ± 11*	362 ± 10*
	100%	181%	192%	188%	187%
5. HPM 500 mg/kg	190 ± 4	246 ± 6b*	212 ± 7b	189 ± 11b	160 ± 10b
	100%	130%	112%	100%	85%
6. HPAp 160 mg/kg	197 ± 5	288 ± 7b*	301 ± 8b*	317 ± 11b*	323 ± 9*
	100%	147%	153%	161%	165%
7. HPBk 218 mg/kg	189 ± 4	336 ± 10*	343 ± 12*	359 ± 9*	351 ± 10*
	100%	178%	181%	190%	186%
8. HPEm 330 mg/kg	191 ± 4	319 ± 14*	314 ± 12b*	319 ± 13b*	327 ± 14*
	100%	168%	166%	169%	173%
9. HPRm 90 mg/kg	194 ± 5	273 ± 14b*	279 ± 12b*	291 ± 11b*	295 ± 12b*
	100%	142%	145%	151%	154%
10. HPSm 80 mg/kg	199 ± 6	314 ± 12*	333 ± 13*	351 ± 10*	350 ± 12*
	100%	158%	168%	178%	178%

The values represent the mean ± SEM. Letter “a” indicates significant difference versus normoglycemic (N) group at *p* < 0.05, letter “b” indicates significant difference versus hyperglycemic + pyruvate group (HP) at *p* < 0.05; * indicates significant difference with time 0 of the same group at *p* < 0.05. Change in blood glucose levels is represented as percentage. N, normoglycemic group; NP, normoglycemic + pyruvate group; H, hyperglycemic; HP, hyperglycemic + pyruvate group; HPM, hyperglycemic + pyruvate + metformin group; HPAp, hyperglycemic + pyruvate + *Ageratina petiolaris* group; HPBk, hyperglycemic + pyruvate + *Bromelia karatas* group; HPEm, hyperglycemic + pyruvate + *Equisetum myriochaetum* group; HPRm, hyperglycemic + pyruvate + *Rhizophora mangle* group; HPSm, hyperglycemic + pyruvate + *Smilax moranensis* group.

For the treatments, metformin was effective in reducing blood glucose levels (HPM group; Table 1) since it was able to attenuate the rising glucose observed at 30 min in the HP group by approximately 51%. In addition, metformin had a significant hypoglycemic effect over the next 1 h and 30 min by decreasing the glucose levels beyond the basal level of the HPM group (15% lower).

The plant extracts did not completely block the rising levels of glucose by pyruvate administration since the basal glucose values were significantly lower than those at 30 min in all experimental groups. Although all of the extracts were able to reduce the hyperglycemic peak, only two plants showed a significant decrease. The experimental groups treated with the extracts of *A. petiolaris* and *R. mangle* exhibited similar behavior (HPAp and HPRm groups). These plant extracts were able to significantly reduce the hyperglycemic peak by approximately 20% at 30 min versus the HP control group. This significant difference was maintained during the remainder of the test, showing an inhibitory effect on gluconeogenesis.

The *S. moranensis* extract (HPSm group) decreased the hyperglycemic peak by 11%; however, the glucose values started to increase starting at 60 min. Similarly, the HPEm group reduced its hyperglycemic peak by approximately 10%; nevertheless, the *E. myriochaetum* extract maintained significantly lower glucose levels after 60 and 90 min. These outcomes showed that both extracts, at the doses used, exhibited a weak inhibitory effect on gluconeogenesis: the former lowered the hyperglycemic peak, and the latter showed an antihyperglycemic effect. No significant differences were found in gluconeogenesis after oral administration of the *B. karatas* extract (HPBk group) versus the HP control group at any time point; therefore, this extract had no effect on glucose synthesis generated by pyruvate.

In addition to the comparation among extract effects on the pyruvate tolerance test (Figure 1A), an AUC analysis was performed for the evaluation of the effect of the plant extracts on blood glucose at a global level (Figure 1B). This analysis showed that *B. karatas* and *S. moranensis* had no effect on the overall glucose generated by pyruvate injection since their AUC values were not statistically significant versus the AUC of HP. However, *A. petiolaris*, *E. myriochaetum*, and *R. mangle* significantly decreased whole glucose levels over 120 min versus the HP group. Among these three extracts, *R. mangle* showed the greatest hypoglycemic effect due to gluconeogenesis inhibition by pyruvate administration since this AUC was significantly different from the others. In contrast to the extracts, the AUC of metformin was notably similar to the H group; that is, this hypoglycemic drug was able to restore glucose to the levels prior to pyruvate administration.

To summarize, administration of *A. petiolaris* and *R. mangle* extracts showed both higher and more stable HGO inhibition than *B. karatas*, *E. myriochaetum*, and *S. moranensis*.

### *In vitro* G6Pase Activity

**FIGURE 2:**
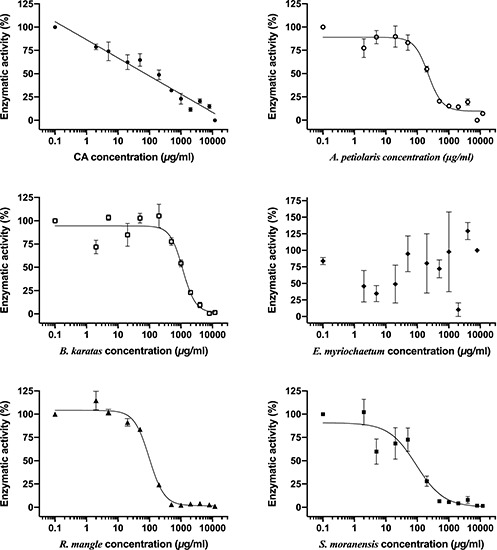
Comparison of inhibitory concentration-response curves of chlorogenic acid and each plant extract on G6Pase system activity. Each point represents the mean of three replicates ± SEM. CA, chlorogenic acid.

To evaluate the direct inhibition of G6Pase activity, concentration-response assays were performed, and the best-fitting regression model (linear or non-linear) to obtain the IC_50_ of each plant extract was chosen. CA was used as a positive control since it is a well-characterized inhibitor of G6Pase T1 translocase. As shown in Table 2 and Figure 2, CA exerted the most potent inhibitory effect, followed by the extracts from *S. moranensis* and *R. mangle*. *A petiolaris* was the less effective; however, it exhibited a higher inhibitory effect than *B. karatas*. On the other hand, *E. myriochaetum* did not show an effect at any assayed concentration.

**TABLE 2:** IC_50_ values of plant extracts obtained from G6Pase system inhibition assay.

**Plant extract**	**IC_50_ value**
1. CA	63 μg/ml
2. *A. petiolaris*	223 μg/ml
3. *B. karatas*	1136 μg/ml
4. *E. myriochaetum*	–
5. *R. mangle*	99 μg/ml
6. *S. moranensis*	84 μg/ml

## Discussion

In Mexican traditional medicine, a common practice to treat T2D among patients is to drink an infusion of a medicinal plant between meals (fasting state) to control the disease (blood glucose levels). The fact that the plants are consumed in the fasting state associated with the statement that CA and other polyphenols have been related with the blockage of the enzyme G6Pase (because these compounds have been reported in several plants) led us to propose a link between the traditional use and the action mechanism. This link is not necessarily the best pharmacological option, but it explains the traditional use, considering the great number of plants that have been reported as hypoglycemic.

According to our results, the pyruvate-STZ-NA model was a good tool to assess the effect of plant extracts on HGO *in vivo* since pyruvate administration raised and maintained the glucose levels of fasting animals to approximately 200 mg/dl throughout the test. Additionally, the model was responsive to the hypoglycemic drug metformin. As shown, the glucose levels of the N group returned to a basal value at the end of the test because of an unaltered insulin secretion. On the other hand, the H group showed an increase in blood glucose, which never returned to the initial values due to impaired insulin secretion, resulting in a low uptake of glucose by skeletal muscle and adipose tissue. Despite the damage to insulin secretory cells caused by STZ, the main effect of metformin was shown: its ability to reduce glucose levels via inhibition of gluconeogenesis (Tan et al., 2016).

**FIGURE 3:**
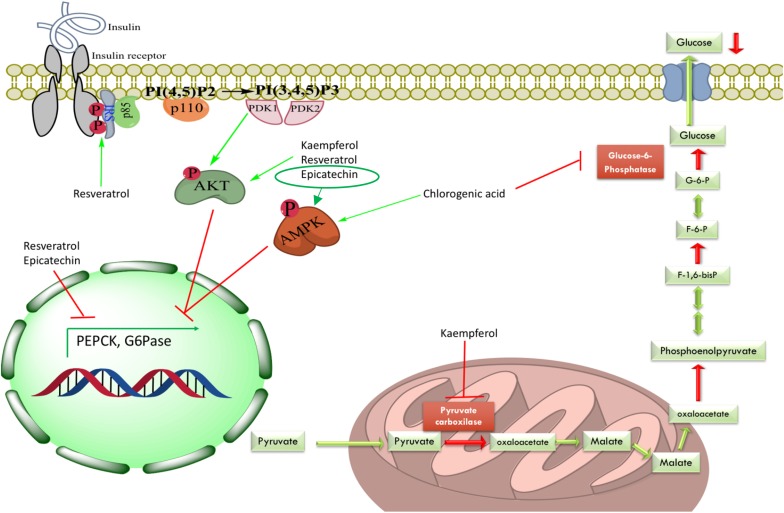
Mechanisms of hepatic gluconeogenesis inhibition exerted by some identified compounds from evaluated plant extracts in this study. Active compounds could have two main ways to decrease HGO: (1) direct inhibition of gluconeogenic enzymes, for instance, inhibition of G6Pase system by CA and inhibition of PC by kaempferol; or (2) decreasing expression levels of gluconeogenic enzymes via Akt pathway (kaempferol, resveratrol and, epicatechin) or AMPK activation (CA and epicatechin). IRS, insulin receptor substrate; PI(4,5)P2, phosphatidylinositol 4,5-bisphosphate; PI(3,4,5)P3, phosphatidylinositol 3,4,5-triphosphate; PDK1, phosphoinositide-dependent kinase 1; PDK2, phosphoinositide-dependent kinase 2; Akt, protein kinase B; AMPK, AMP-activated protein kinase; G6P, glucose-6-phosphate; F-6-P, fructose-6-phosphate; F-1,6-bisP, fructose-1,6-bisphosphate; G6Pase, glucose-6-phosphatase.

The tested plant extracts were able to decrease the hyperglycemic peak after pyruvate administration. Based on the phytochemical composition of the extracts and previous reports in the international literature (see below), possible explanations for the observed effects could be: (1) the presence of CA in the extracts, which was not only able to inhibit G6Pase T1 translocase but also able to raise the phosphorylation levels of AMP-activated protein kinase (AMPK) (Ong et al., 2013); (2) inhibition of the G6Pase system by other compounds; (3) inhibition of another gluconeogenic enzyme; or (4) suppression of HGO via protein kinase B (Akt) or AMPK activation (Figure 3).

*Rhizophora mangle* was able to effectively inhibit the G6Pase system *in vitro*. In addition, the reduction of gluconeogenesis *in vivo* by the *R. mangle* extract can also be attributed to the decrease in the activity of other gluconeogenic enzymes such as PEPCK. Epicatechin, one of the main compounds found in *R. mangle*, has been shown to reduce the enzyme expression leading to diminished HGO in both NRK-52E and HepG2 cells. Additionally, this phenolic compound increased the total and phosphorylated protein levels of the insulin receptor (IR), insulin receptor substrate-1 (IRS-1), and AMPK (Cordero-Herrera et al., 2014; Álvarez-Cilleros et al., 2018).

*Ageratina petiolaris* showed an inhibitory effect in the pyruvate tolerance test *in vivo* that could be correlated with its capacity of decreasing the activity of G6Pase system *in vitro* owing to its content of CA (Bustos-Brito et al., 2016). Furthermore, D-chiro-inositol, a polyalcohol present in many Fabaceae plants that has been chronically administered to high-fat diet STZ-treated Sprague Dawley rats, has induced a significant decreasing in fasting insulin levels, increasing hepatic glycogen, raising expression levels of glycogen synthase and GLUT4 genes, and increasing phosphorylation of hepatic Akt (Gao et al., 2016). Therefore, the isomer L-chiro-inositol identified in *A. petiolaris*, by our group, could have a similar ability to positively modulate the insulin signaling pathway, which would inhibit HGO in the pyruvate tolerance test or promote glycogen synthesis.

*Smilax moranensis* showed the most potent inhibitory effect on the G6Pase system, this could also be correlated with the content of CA and the presence of 3-O-caffeoylquinic acid, an isomer of CA (Romo-Pérez et al., 2019). However, it did not show a significant effect in the pyruvate tolerance test, which could be explained by its pharmacokinetics, either poor intestinal absorption (Ziberna et al., 2014) or metabolism of the G6Pase inhibitor before it can reach the target. *S. moranensis* contains *trans*-resveratrol, which has been associated with increased phosphorylation of both Akt and IRS-1; this activation involves the reduction of both insulin resistance and gluconeogenic enzyme expression (Szkudelski and Szkudelska, 2015).

*Equisetum myriochaetum* had a positive effect over the HGO in the pyruvate tolerance test but did not show G6Pase system inhibition, thus its mechanism of action could be related to directly inhibit another gluconeogenic enzyme. Some of the main isolated metabolites from *E. myriochaetum* were several types of kaempferols that have been shown to decrease pyruvate carboxylase (PC) activity with no change in protein expression levels due to an increase in hepatic Akt in high-fat diet-fed obese mice (Alkhalidy et al., 2018).

Although *B. karatas* showed a hypoglycemic effect in previous studies (Andrade-Cetto and Medina-Hernández, 2013), its aqueous extract did not show an inhibitory effect in the pyruvate tolerance test. Moreover, in the G6Pase inhibition assay, the IC_50_ for this extract was the highest one, suggesting that the mechanism of action of this extract is not related to the inhibition of HGO. However, further studies must be performed to determine how this hypoglycemic plant works.

Inhibition of G6Pase, which has been proposed to be a shared hypoglycemic mechanism of plants, presents two major problems as a therapeutic target in patients with T2D; (1) since this enzyme is present in both gluconeogenesis and glycogenolysis pathways, inhibitors may cause hypoglycemia; and (2) accumulation of intracellular G6P may induce the expression of lipogenic genes that could result in hepatic steatosis (Agius, 2007). Further studies are needed to prove if the extracts affect HGO in other ways, but a possible common mechanism of action for these plants could be found.

## Conclusion

In summary, four of the tested plants showed an inhibitory effect on HGO by either decreasing gluconeogenesis in the pyruvate tolerance test or diminishing the HGO-related activity of G6Pase. This inhibition could be a shared mechanism that could be associated with the phytochemical composition of the plants, CA among other compounds, contributing to their hypoglycemic effect. These observations can be correlated to the traditional way of consumption of the plants.

The present work was a starting point approach to characterize the hypoglycemic effects related to HGO inhibition. Based on the known plant composition and literature review, further studies must be performed to test whether these extracts have an effect not only on other gluconeogenic enzymes but also on the signal transduction pathways related to gluconeogenesis.

## Data Availability Statement

All datasets generated for this study are included in the article/Supplementary Material.

## Ethics Statement

The protocol was approved by the Committee of Academic Ethics and Scientific Responsibility (CEARC) of the Faculty of Sciences, UNAM.

## Author Contributions

AA-C idealized the work. All authors listed have made a substantial, direct and intellectual contribution to the work, and approved it for publication.

## Conflict of Interest

The authors declare that the research was conducted in the absence of any commercial or financial relationships that could be construed as a potential conflict of interest.
