# Supplementary material for: Hepatic Glucose Output Inhibition by Mexican Plants Used in the Treatment of Type 2 Diabetes
Source: Front Pharmacol. 2020 Mar 3;11:215. doi: 10.3389/fphar.2020.00215 (PMC7065531; doi:10.3389/fphar.2020.00215)
Supplement: Supplementary file 1 [file Data_Sheet_1.pdf]

Chromatographic profiles and previous isolated compounds of the tested plants.

Chromatographic conditions:

High performance liquid chromatography (HPLC) was carried out in an Agilent 1260 HPLC instrument, equipped with an Agilent G1315C UV diode array detector (DAD). Chromatographic profile elaboration was performed with a Phenomenex (Luna Omega Polar C_{18}, 50 × 2.1 mm id., 1.6 μm) reverse phase column. Elution was carried out as described in table S1, the column temperature was kept at 35ºC. System control, data collection and data processing were accomplished using OpenLAB LC 1260 chromatography software. Working solutions of samples were prepared dissolving 10.0 mg of test sample in 1 mL of the water and (3 μL) were injected with an autosampler. For UV detection, the wavelength program was set at an acquisition of λ 230, 254, 280, 320 and 365 nm; 320 and 280 nm were selected as the optimum wavelength for the extracts.

Table S1. Chromatographic conditions

Time (min)	A[%]	B[%]	C[%]	Flow rate (mL/min)
0	100	0	0	0.35
10	84	1	15	0.35
20	84	1	15	0.35
30	69	1	30	0.35
37	0	5	95	0.35
37.5	0	5	95	0.35
40	100	1	0	0.35

A: 0.1% formic acid, B: methanol, C: acetonitrile

Ageratina petiolaris (Moc. ex DC.) R.M. King & H. Rob.

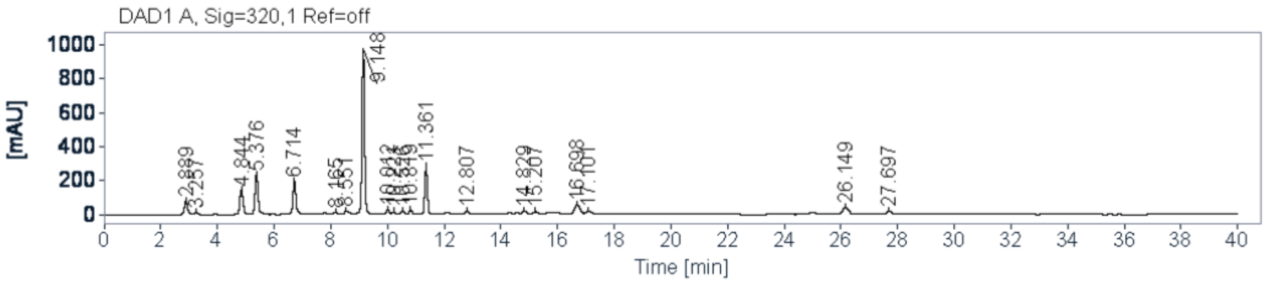

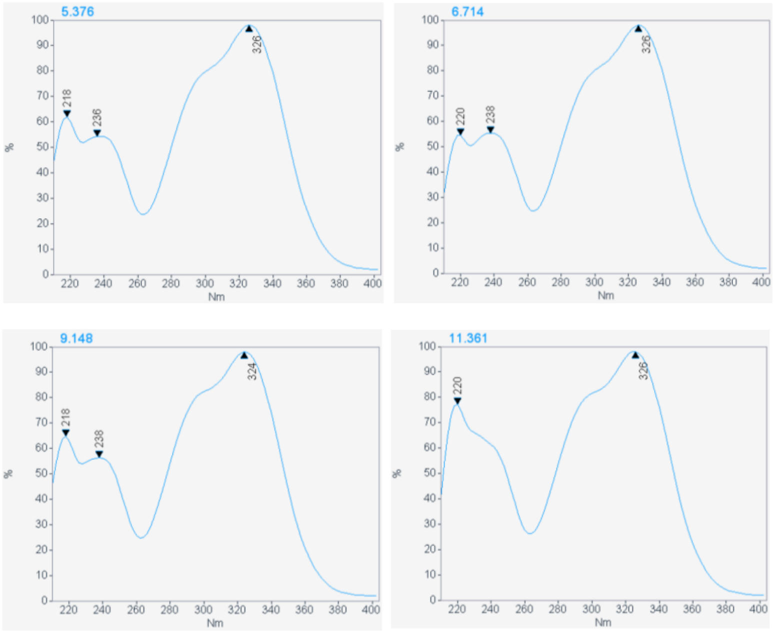

Figure S2. Chromatographic profile of the aqueous extract of *A. Petiolaris.* Upper; DAD spectra, lower; full uv of the main peaks.

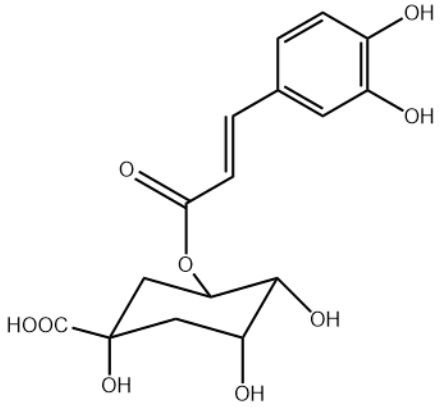

1

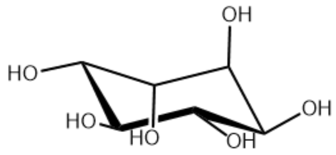

2

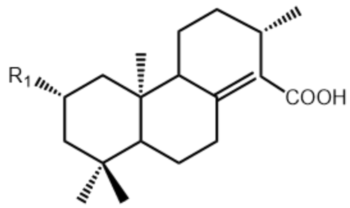

	R_1
**3**	O-iso-valeroyl
**8**	O-tigloyl

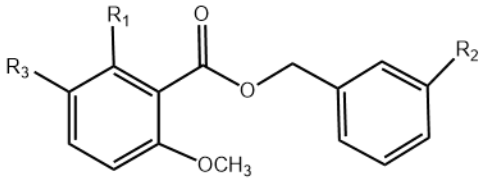

	R_1	R_2	R_3
**4**	OH	H	H
**5**	OCH_3	H	H
**6**	OCH_3	OCH_3	H
**7**	OH	H	OCH_3

The uv spectra present similar compounds, the majority is chlorogenic acid and the other main compounds seems to be chlorogenic derivatives. The phytochemical composition was reported in; (Bustos-Brito *et al.* 2016).

Bromelia karatas L.

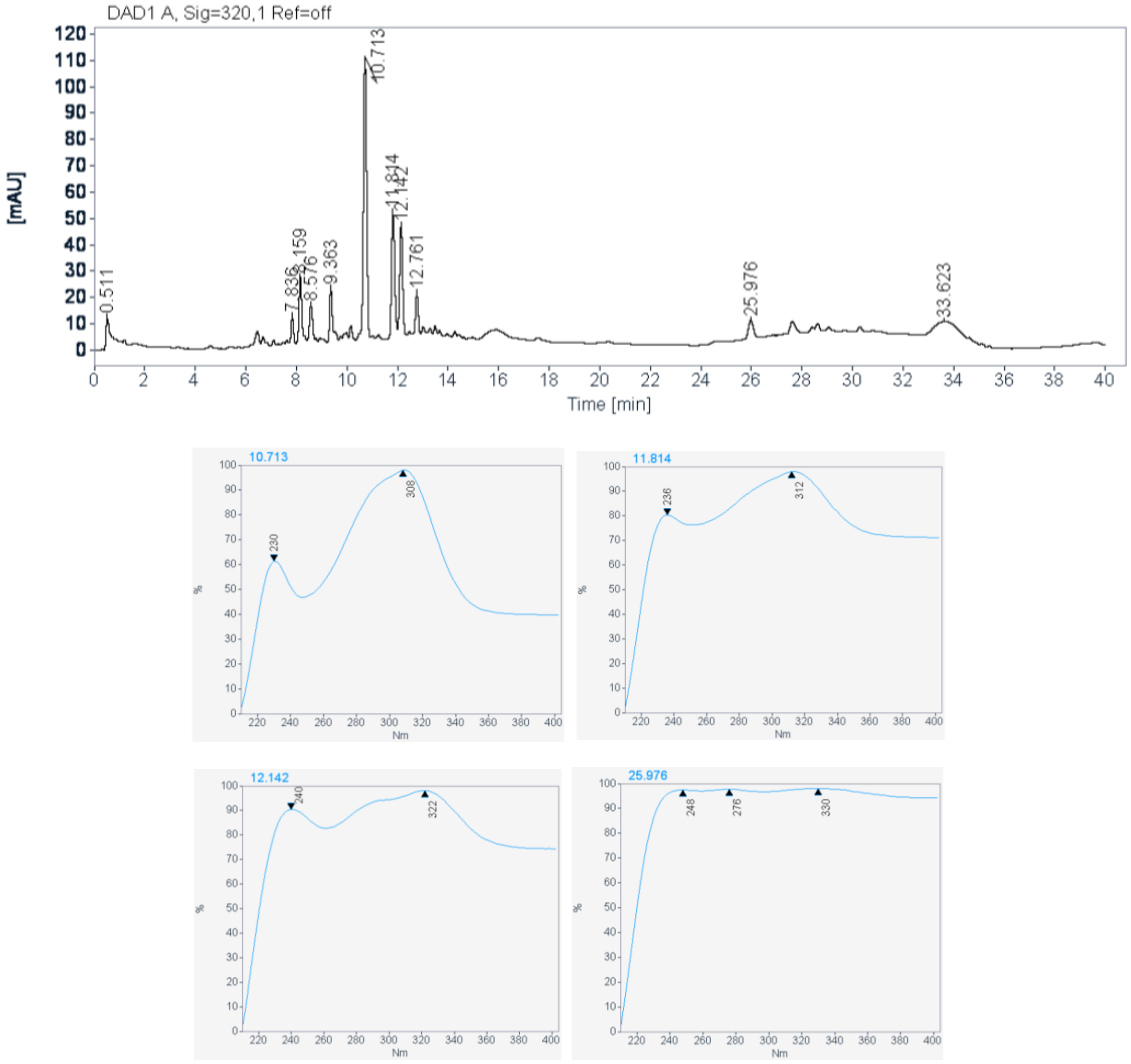

Figure S3. Chromatographic profile of the aqueous extract of *B. Karatas*. Upper; DAD spectra, lower; full uv of the main peaks.

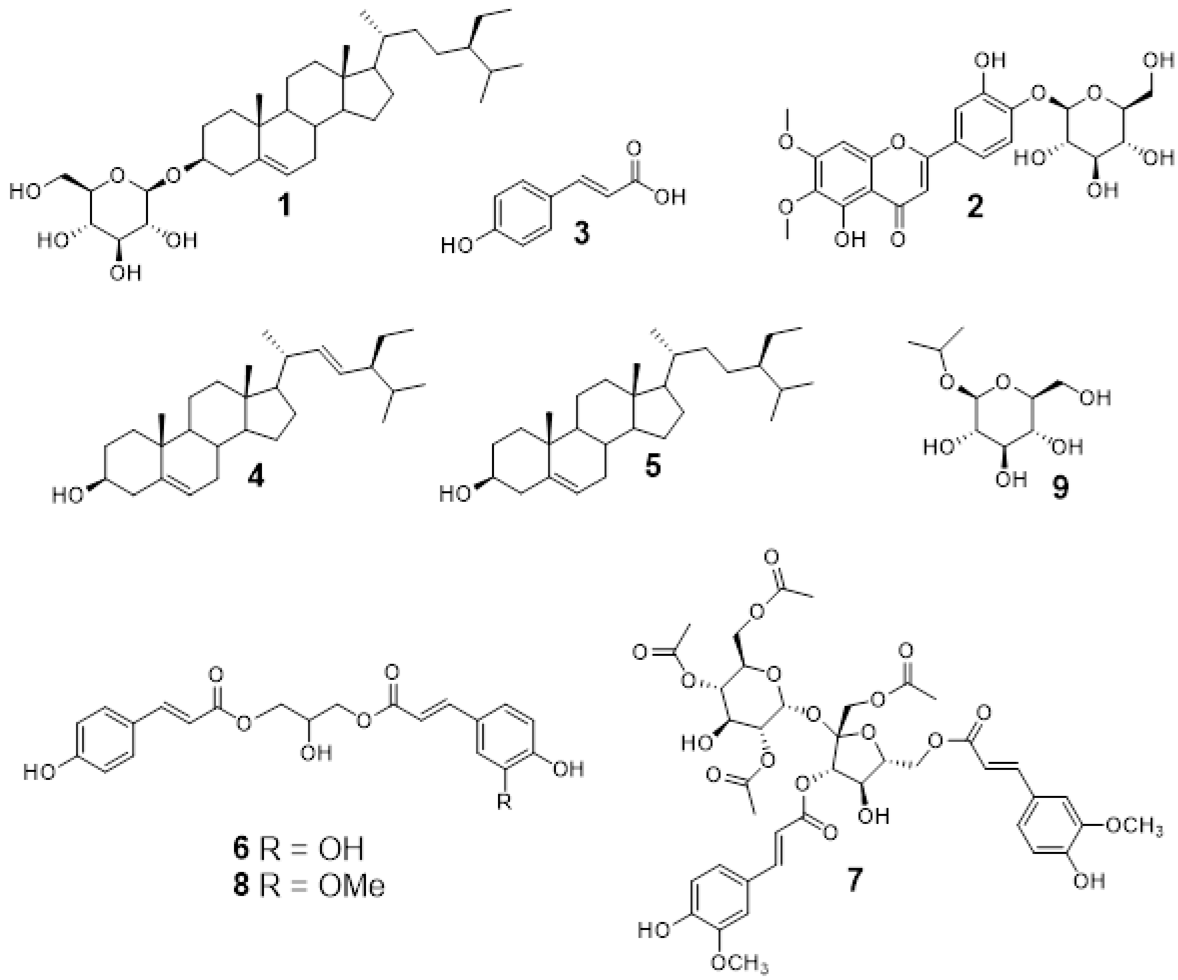

Bromelia presents uv spectra like compounds derived from cinnamic acid and flavonoids as minor compounds. The phytochemical composition was reported in; (Escandón-Rivera *et al*. 2019).

Equisetum myriochaetum Schltdl. & Cham.

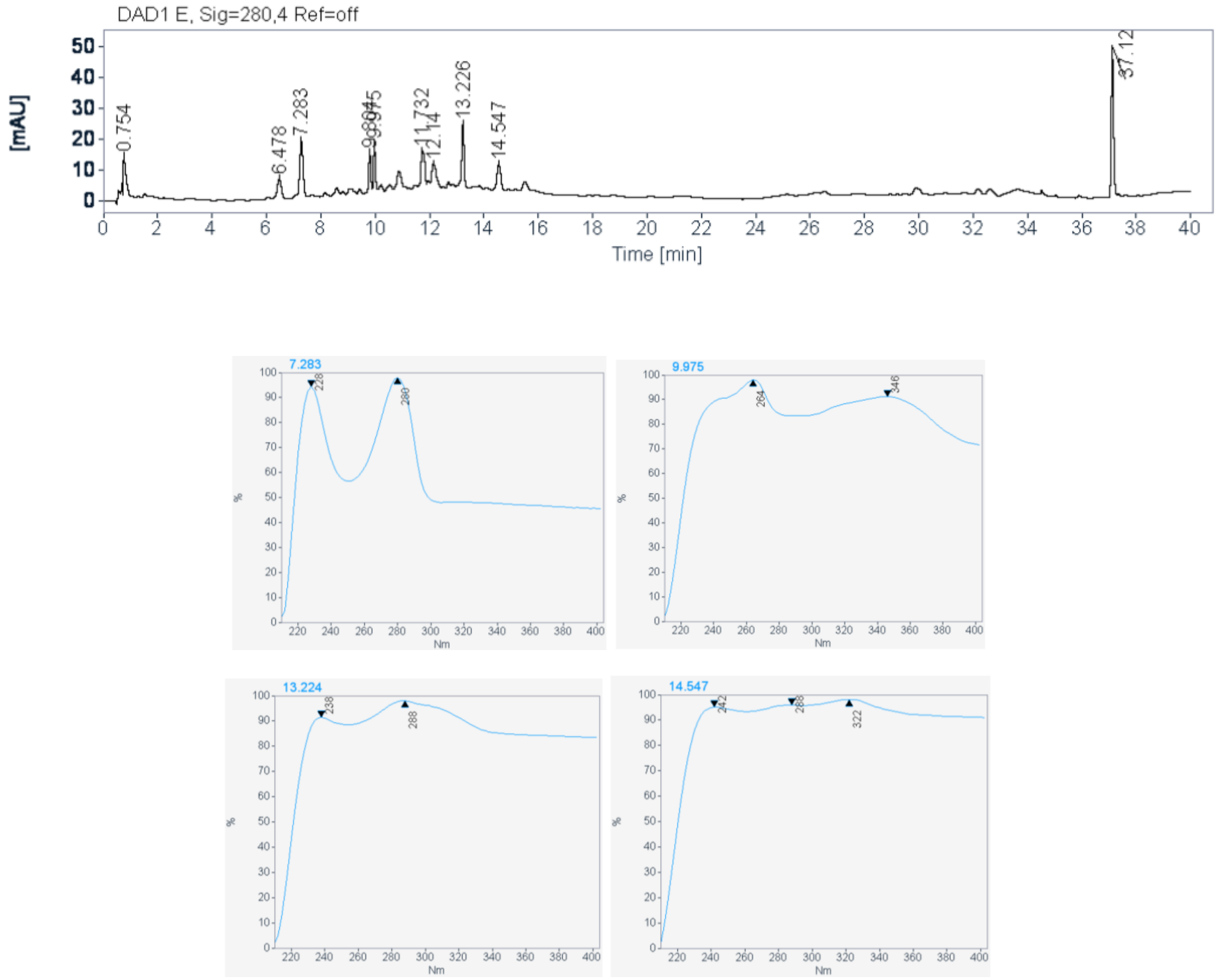

Figure S4. Chromatographic profile of the aqueous extract of *E. myriochaetum*. Upper; DAD spectra, lower; full uv of the main peaks.

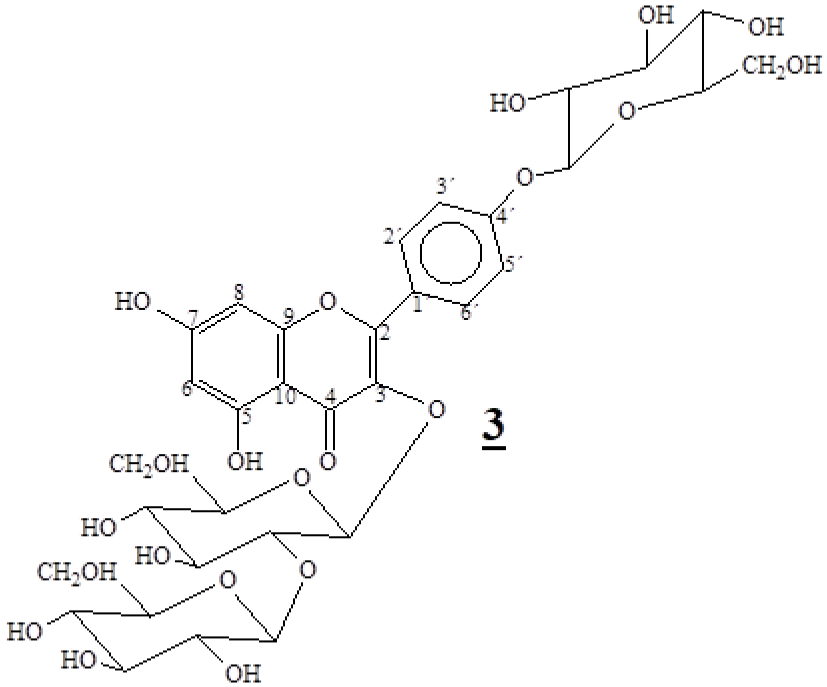

3

The main components are flavonol glycosides. The phytochemical composition was reported in; (Widenfeld and Andrade-Cetto, 2000).

Rhizophora mangle L.

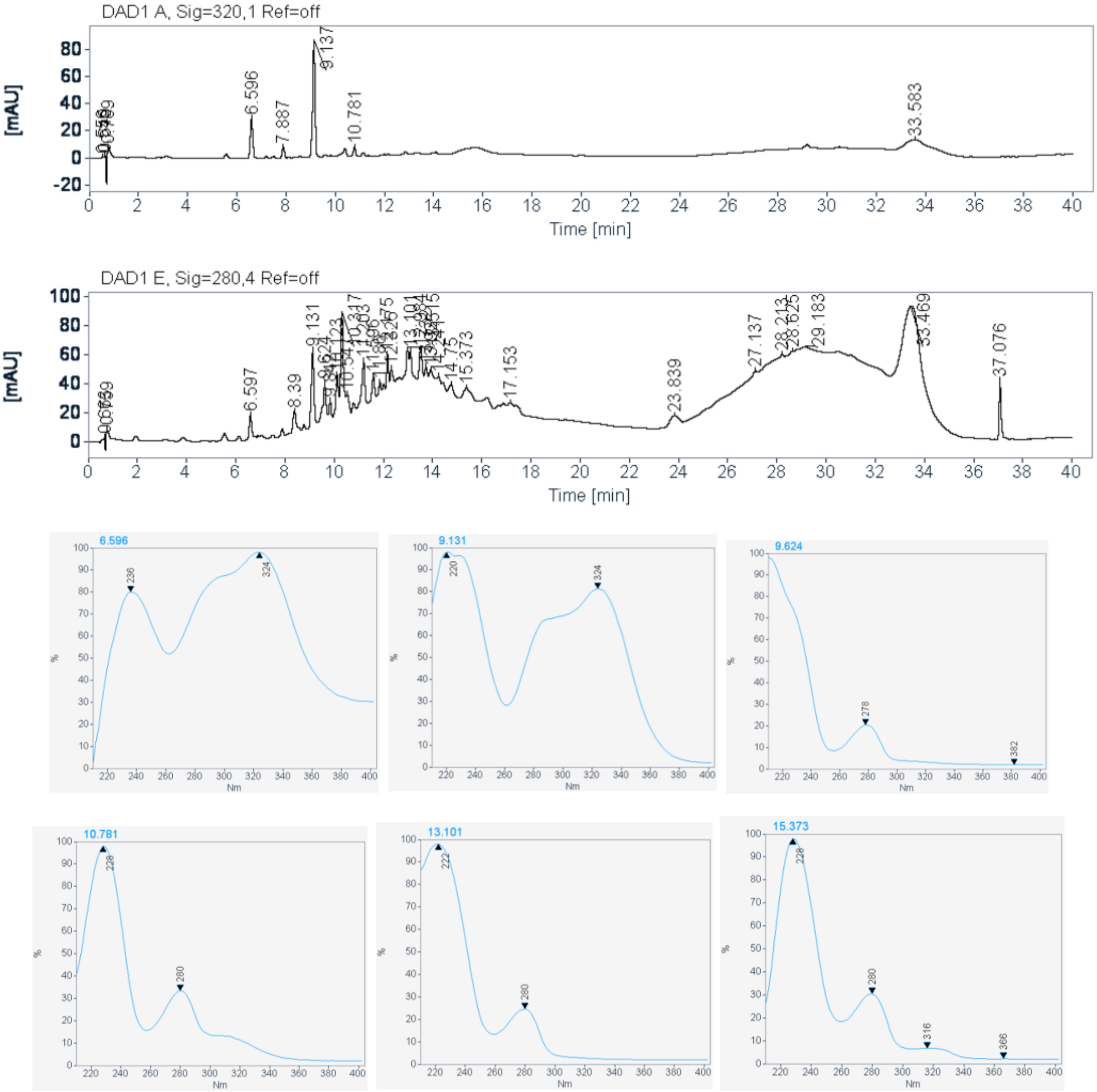

Figure S5. Chromatographic profile of the aqueous extract of *R. mangle*. Upper; DAD spectra, lower; full uv of the main peaks.

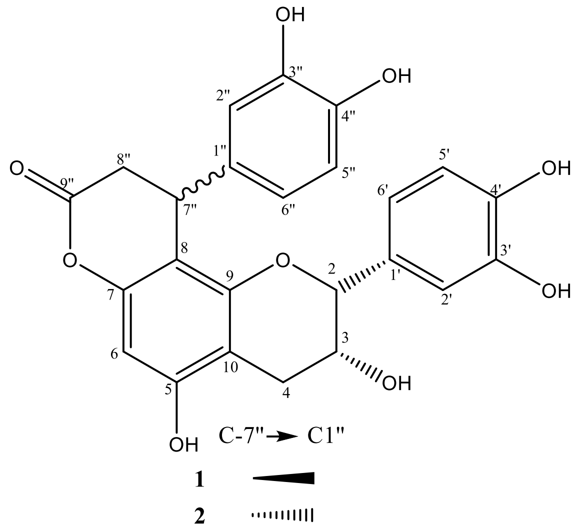

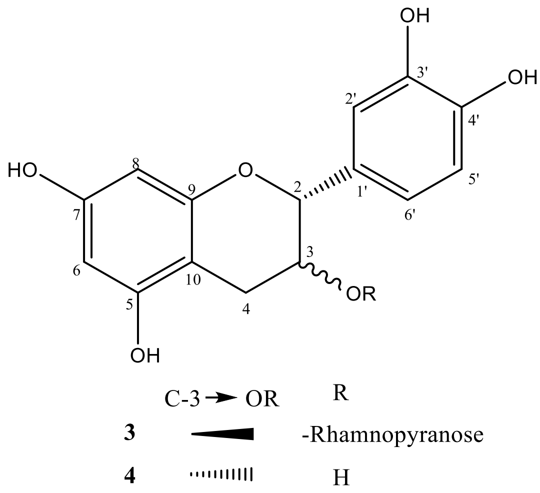

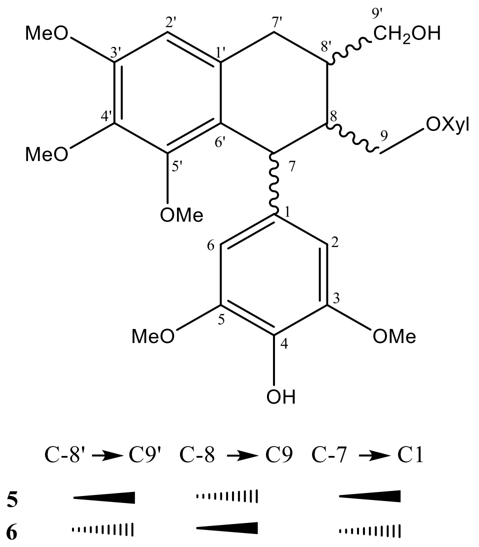

The main components are flavanols and anthocyanins. The phytochemical composition was reported in; (Andrade-Cetto, A., *et al.*, 2017).

Smilax moranensis M. Martens & Galeotti

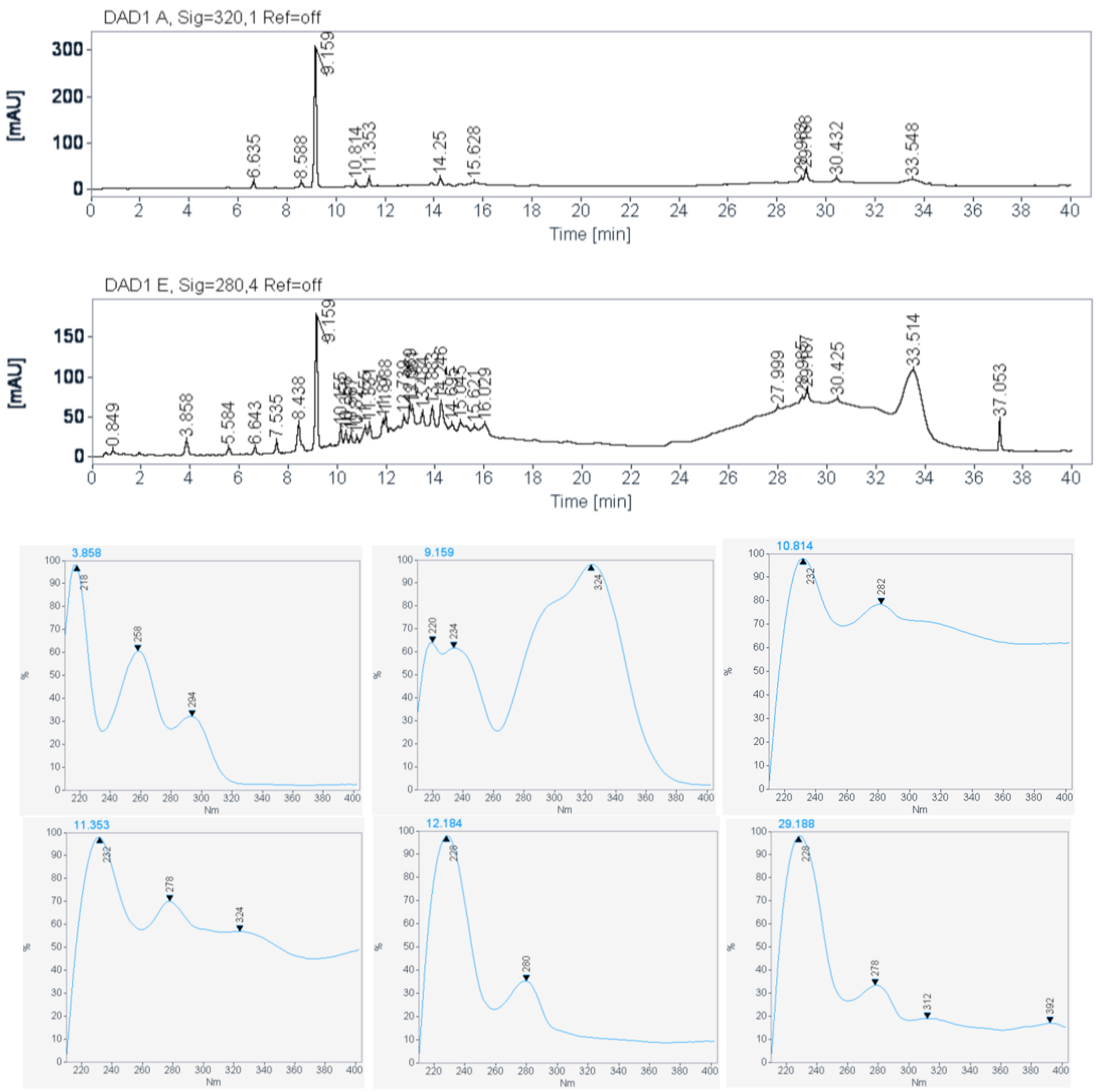

Figure S6. Chromatographic profile of the aqueous extract of S. moranensis. Upper; DAD spectra, lower; full uv of the main peaks.

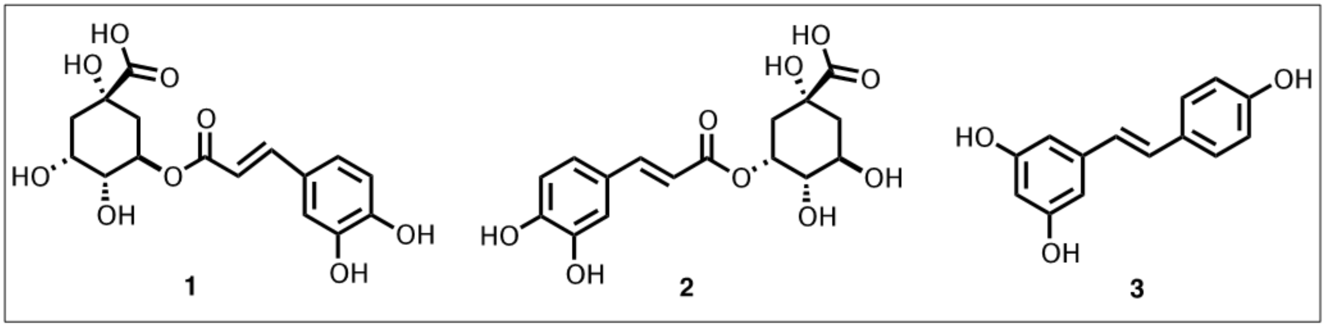

The main components are flavanols, chlorogenic acid derivates and anthocyanins. The phytochemical composition was reported in; (Romo-Pérez, *et al*. 2019).
