# Supplementary material for: Hepatic Glucose Output Inhibition by Mexican Plants Used in the Treatment of Type 2 Diabetes
Source: Front Pharmacol. 2020 Mar 3;11:215. doi: 10.3389/fphar.2020.00215 (PMC7065531; doi:10.3389/fphar.2020.00215)
Supplement: Supplementary file 2 [file Data_Sheet_2.pdf]

Table S2: Studied plants and extracts

Plant/Voucher	Extract-dose	Reference/ Plant determination	Extract-dose selected
Rhizophora mangle L. IMMSM15816	Water – 5.9 mg/kg	(Andrade-Cetto and Rubalcaba-Mares, 2012)* Andrade-Cetto A.	
	Water – 59 mg/kg		
	Ethanol-water – 9 mg/kg		
	Ethanol-water – 90 mg/kg		*
Smilax moranensis M.Martens & Galeotti IMSS 15815	Water – 20 mg/kg	(Andrade-Cetto, 2011)* Ramiro Cruz Duran	
	Water – 200 mg/kg		
	Ethanol-water – 8 mg/kg		
	Ethanol-water – 80 mg/kg		*
Ageratina petiolaris (Moc. & Sessé ex DC.) R.M.King & H.Rob MEXU-1333471	Water - 40 mg/kg	(Bustos-Brito et al., 2016)* Jose LuisVillaseñor	
	Water - 160 mg/kg		*
	Methanolic - 67 mg/kg		
	Methanolic - 268 mg/kg		
Bromelia karatas L. IMMSM15814 Syn. B. plumieri	Water – 35 mg/kg	(Andrade-Cetto and Medina-Hernández, 2013)* Ramiro Cruz Duran	
	Water – 350 mg/kg		
	Ethanol-Water – 30 mg/kg		
	Ethanol-Water – 300 mg/Kg		
	Water – 218 mg/kg	(Escandón-Rivera et al., 2019)	*
Equisetum myriochaetum Schltdl. & Cham. IMMSM 14689	Water (patients) – 330 mg/kg	(Revilla et al., 2002)* Andrade-Cetto A.	*

Doses tested in previous works, the * in the reference indicates the first description of the plant.
